# Evidence that Adaptation in *Drosophila* Is Not Limited by Mutation at Single Sites

**DOI:** 10.1371/journal.pgen.1000924

**Published:** 2010-06-17

**Authors:** Talia Karasov, Philipp W. Messer, Dmitri A. Petrov

**Affiliations:** Department of Biology, Stanford University, Stanford, California, United States of America; Fred Hutchinson Cancer Research Center, United States of America

## Abstract

Adaptation in eukaryotes is generally assumed to be mutation-limited because of small effective population sizes. This view is difficult to reconcile, however, with the observation that adaptation to anthropogenic changes, such as the introduction of pesticides, can occur very rapidly. Here we investigate adaptation at a key insecticide resistance locus (*Ace*) in *Drosophila melanogaster* and show that multiple simple and complex resistance alleles evolved quickly and repeatedly within individual populations. Our results imply that the current effective population size of modern *D. melanogaster* populations is likely to be substantially larger (≥100-fold) than commonly believed. This discrepancy arises because estimates of the effective population size are generally derived from levels of standing variation and thus reveal long-term population dynamics dominated by sharp—even if infrequent—bottlenecks. The short-term effective population sizes relevant for strong adaptation, on the other hand, might be much closer to census population sizes. Adaptation in *Drosophila* may therefore not be limited by waiting for mutations at single sites, and complex adaptive alleles can be generated quickly without fixation of intermediate states. Adaptive events should also commonly involve the simultaneous rise in frequency of independently generated adaptive mutations. These so-called soft sweeps have very distinct effects on the linked neutral polymorphisms compared to the standard hard sweeps in mutation-limited scenarios. Methods for the mapping of adaptive mutations or association mapping of evolutionarily relevant mutations may thus need to be reconsidered.

## Author Summary

Adaptation in eukaryotes is often assumed to be limited by the waiting time for adaptive mutations. This is because effective population sizes are relatively small, typically on the order of only a few million reproducing individuals or less. It should therefore take hundreds or even thousands of generations until a particular new mutation emerges. However, several striking examples of rapid adaptation appear inconsistent with this view. Here we investigate a showpiece case for rapid adaptation, the evolution of pesticide resistance in the classical genetic organism *Drosophila melanogaster*. Our analysis reveals distinct population genetic signatures of this adaptation that can only be explained if the number of reproducing flies is, in fact, more than 100-fold larger than commonly believed. We argue that the old estimates, based on standing levels of neutral genetic variation, are misleading in the case of rapid adaptation because levels of standing variation are strongly affected by infrequent population crashes or adaptations taking place in the vicinity of neutral sites. Our results suggest that many standard assumptions about the adaptive process in eukaryotes need to be reconsidered.

## Introduction

The speed of adaptation in eukaryotes is commonly assumed to be limited by the waiting-time for an appropriate adaptive mutation. This notion is based on estimates of the population parameter Θ = 4*N_e_μ* (the product of effective population size *N_e_* and per-site mutation rate *μ*) derived from levels of standing neutral variation. Θ can be interpreted as the rate at which new mutations arise in the population [1]. In contrast to many prokaryotes or viruses, where Θ can easily be on the order of one or larger - and consequently most single nucleotide mutations exist in the population at every given time – estimated values of Θ in eukaryotes are typically much smaller than one [1]. Adaptation should thus be substantially retarded, especially when adaptive alleles need to carry several independent mutations.

However, adaptation to anthropogenic changes such as the evolution of insecticide resistance has been observed to occur very rapidly and often involves complex alleles [2]–[7]. One possible explanation for such cases of rapid adaptation is that complex resistant alleles predate environmental changes [8], [9]. The other possibility is that adaptive mutations emerge more quickly in eukaryotic populations than commonly believed. The latter would imply that estimates of Θ have to be reconsidered in the context of rapid adaptation.

In order to understand the population parameters that allow for rapid adaptation in eukaryotes, we study here a well-documented example: the evolution of pesticide resistance in *D. melanogaster*.

Acetylcholinesterase (AChE), a key neuronal signalling enzyme, is the major target of the most commonly used insecticides, organophosphates (OPs) and carbamates (CMs) [10]. Introduced in the 1950–1960's, these insecticides have been used pervasively around the world since then. Within a few years of their introduction cases of insecticide-resistant AChE alleles emerged [11] and today insecticide-resistant AChE has been observed and characterized in numerous arthropod species [2]–[7].

In *D. melanogaster*, four particular point mutations at highly conserved sites (I161V, G265A, F330Y, G368A) of *Ace* (the gene coding for AChE) lead to resistance to OPs and CMs [5], [12] (Figure S1). Alleles carrying these mutations singly and in combination have been found in natural populations worldwide [12]. In the presence of OPs, these mutations confer semi-additive resistance: single mutations provide moderate levels of resistance to ∼75% of OPs, any two mutations in combination provide higher levels of resistance to ∼80% of OPs, while alleles with three or four mutations lead to strong resistance to practically all OPs [12]. One 3-mutation allele (I161V, G265A, F330Y) was found worldwide at particularly high frequencies and is a key determinant of resistance to OPs [12]. In the absence of pesticides all resistant alleles are strongly deleterious with the selective coefficient on the order of negative 5–20% [13], [14].

Here we collect data and provide quantitative arguments (both analytical and simulation-based) that the observed signatures of adaptation at *Ace* imply a much larger (∼100-fold or more) effective population size than is commonly assumed for *D. melanogaster*. We discuss the implications of our results for the study of adaptation in Drosophila and other species with large census sizes.

## Results

### Fast and repeated evolution of simple and complex resistance alleles within individual subpopulations of *D. melanogaster*

*D. melanogaster* evolved in sub-Saharan Africa (AF) and spread worldwide over the past 10–16 thousand years [15]. The worldwide spread was associated with a severe bottleneck that resulted in sub-sampling of AF diversity by the out-of-Africa strains [15]. Resistant alleles found outside of AF may either have arisen *in situ* in the derived out-of-Africa populations or were present in the AF population prior to the bottleneck (similar to [8], [9]). These two hypotheses can be distinguished by studying haplotype backgrounds of the resistant alleles. Resistant mutations that evolved in derived populations *in situ*, unlike ancient AF resistant alleles, should reside on the background of sensitive haplotypes common in the exposed out-of-Africa populations that passed through the bottleneck.

We collected *D. melanogaster* sequence data (∼1.5 kb covering the known four sites of resistant mutations in *Ace*) from 93 resistant and sensitive strains. We sequenced 9 alleles from the ancestral AF populations, 10 alleles from the derived Eurasian and American populations collected prior to the 1950s (M strains) [16], and 74 alleles from the recently collected (1990–2009) derived populations in North America (NA) and Australia (AUS) (Table S1 and Table S2).

**Figure 1:**
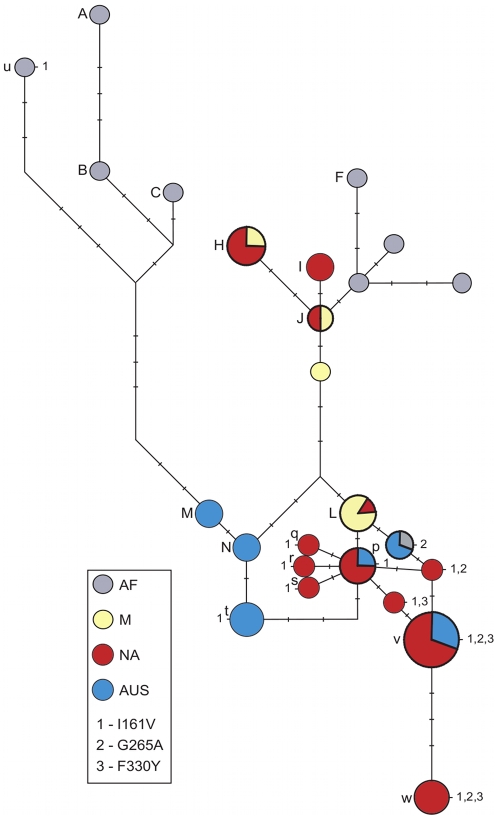
Haplotype network at *Ace*. Alleles containing mutations I161V, G265A and F330Y are numbered 1, 2 and 3 respectively. Sizes of the circles correspond to the number of identical sequences representing each haplotype; tick marks along a branch indicate the number of mutations between two neighbouring haplotypes. Sensitive haplotypes are labelled with capital letters and resistant haplotypes with lowercase letters. Note that our sample is enriched for resistant haplotypes. Resistant NA alleles containing a single mutation (all at the first site) appear to have arisen on the common out-of-Africa haplotype L, with one specific L-related allele (labelled p) present at the highest frequency. The resistant AUS alleles also cluster together. AUS resistant alleles containing a single resistant mutation in the first or second site appear to have arisen either on the background of the common out-of-Africa sensitive haplotype L, or on the background of the specifically AUS haplotype N. The alleles containing two mutations in NA (first plus second or first plus third sites) are all related to the sensitive L haplotype and the common resistant allele (labelled p) containing the mutation in the first site. The 3-mutation alleles are present both in NA and AUS populations (v and w) and are the most closely related to the sensitive L haplotype. There are two resistant alleles containing single mutations in the first and the second site that we detected in AF. One of these is very similar to the AUS alleles containing the second mutation and is likely a migrant from out-of-Africa back to AF. The other appears to have arisen *in situ* in AF (u).

**Figure 2:**
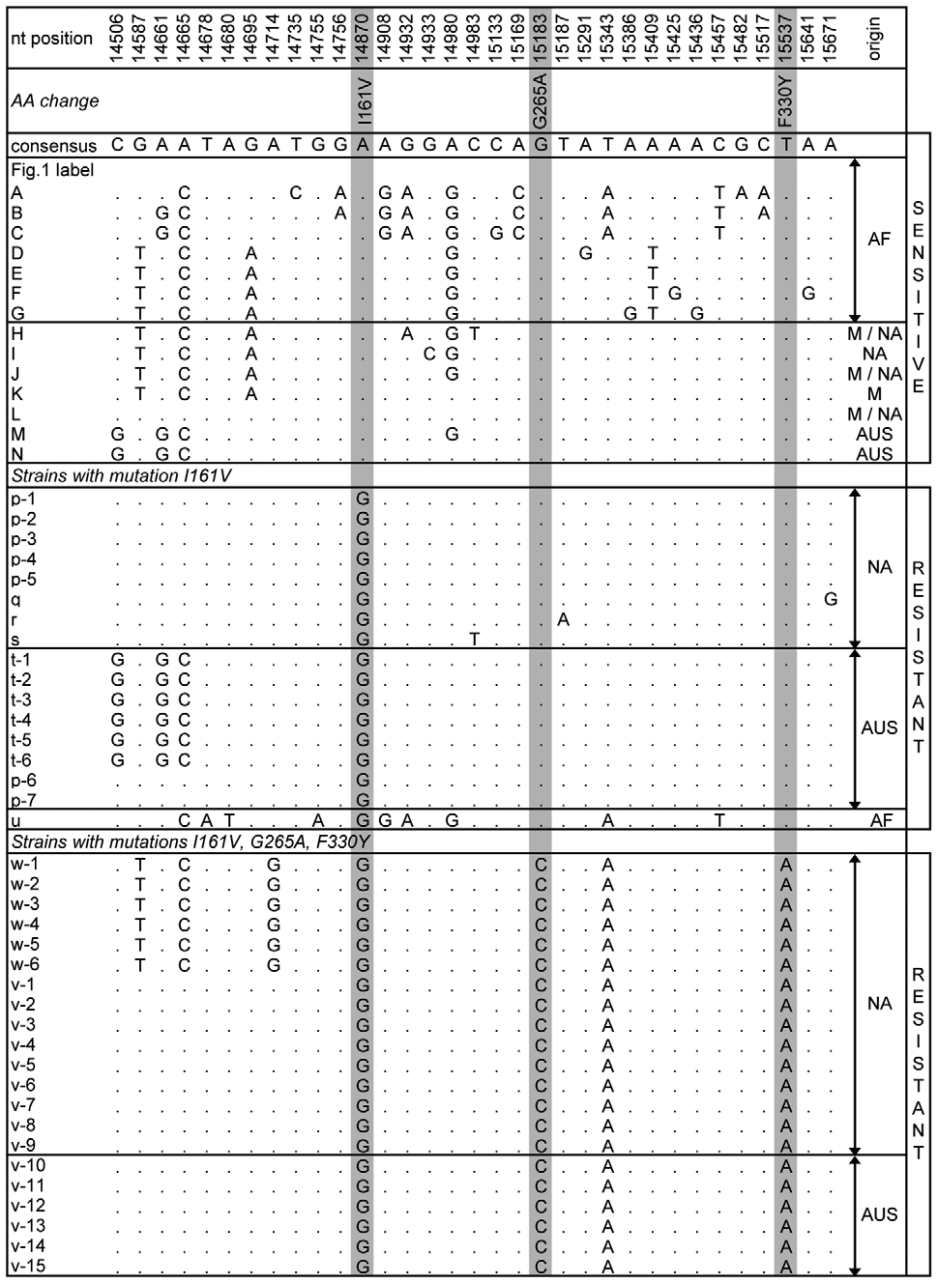
Soft sweeps at *Ace*. The table shows segregating sites within the 1.5-kb region of *Ace*. Each strain is named according to the corresponding letter in Figure 1. When multiple strains shared the same haplotype, they were named with the same letter but with different numbers (*i.e.* w-1, w-2, w-3). For the names and origins of the strains refer to Table S1 and Table S2. The nucleotide position and the consensus sequence at the top of the table correspond to the y^1^; cn^1^ bw^1^ sp^1^ strain. The positions of the three resistant mutations are shaded. The table shows all sensitive haplotypes observed more than once as well as all haplotypes containing the resistant mutation at the first site (I161V) and the ones that contain all three resistant mutations.

We detected resistant mutations at the first three sites (I161V, G265A, F330Y) but did not find the resistant mutation at the fourth site (G368A). We estimated that ∼40% of the strains contain resistant mutations in the modern NA and AUS populations of *D. melanogaster*. Figure 1 shows the most parsimonious haplotype network of the sequenced alleles. Figure 2 shows the segregating sites for sensitive haplotypes, as well as the I161V 1-mutation and the 3-mutation haplotypes (Table S2 shows segregating sites for all sequenced alleles).

In all cases the NA and AUS resistant alleles show no signs of having predated the spread of *D. melanogaster* out-of-Africa. Instead, the resistant alleles appear to have arisen *in situ* in different populations, as indicated by the observation that locally common resistant alleles are present on the locally common sensitive haplotypes. For instance, AUS alleles with the resistant mutation in the first site (marked t) have the haplotype background that is identical to the sensitive haplotype N that is common in AUS but has not been detected by us in NA. In contrast, the NA first site mutation alleles (marked p through s) have the haplotype backgrounds that are nearly identical to the sensitive haplotype L that is common in NA. Additionally, the haplotype background of one of the AF alleles (marked u) with the resistant mutation I161V is substantially diverged from the NA and AUS resistant strains and is more similar to the sensitive alleles common in AF. This suggests a third independent origin of the mutation I161V in AF. Note that the complex 2- and 3-mutation haplotypes also appear to have arisen *in situ* in the derived populations as their haplotype backgrounds are most closely related to the common out-of-Africa sensitive haplotype L.

In summary, the sequence analysis of the resistant and sensitive alleles reveals two signatures of the adaptive evolution of pesticide resistance at the *Ace* gene. First, adaptation has been rapid enough such that in the past 50 years (1000 to at most 1500 generations [17]) multiple resistant alleles including a complex allele containing three independent mutations at three different sites evolved and spread to high frequencies worldwide. Second, many resulting resistant alleles are present on distinct haplotypes that differ in the immediate vicinity of the adaptive sites, such as the adaptive change from A to G at the first site (I161V) in NA and AUS that is located on the haplotypes p, q, r, s, and t (Figure 2).

### Patterns of evolution at *Ace* are inconsistent with small values of Θ: analytical considerations under simple scenarios

Below we consider a simple scenario of a single locus in a panmictic population of effective size *N_e_*. We assume that the resistant alleles were in mutation-selection balance prior to pesticide application with a strongly deleterious selection coefficient of −5% [13], [14] and that they became advantageous after the application of pesticides.

In Box 1 we show that if Θ∼0.01 the probability of successful adaptation from standing genetic variation is less than 1% even if positive selection is extremely strong (*s*∼100%). Thus, if Θ∼0.01, as previously estimated based on analyses of neutral loci in Drosophila [18], [19], we only need to consider the case of adaptation from *de novo* mutations.

**Box 1. Probability of adaptation from standing genetic variation and waiting time for** ***de novo*** **mutation.** Consider a single locus in a panmictic diploid population of constant effective size *N_e_*. New resistant alleles arise at rate Θ*_u_* = Θ/3 (only one out of three mutations give rise to an adaptive allele). Evolution is modelled in a Wright-Fisher infinite alleles framework with selection. Heterozygotes have fitness 1+*s*, fitness is multiplicative, and the locus evolves independently of other loci. Prior to pesticide application resistant alleles are deleterious with selection coefficient *s_d_*<0. The density function *g*(*x*) for the frequency distribution of resistant alleles in mutation-selection balance is then given by [47]

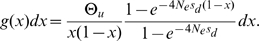
(B1)We thus do not expect resistant alleles to be present in the population most of the time for Θ = 0.01 (*N_e_*∼10^6^) and *s_d_* = −5% because 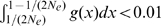.

After the onset of pesticide application, resistant alleles become advantageous (*s*>0). The probability of successful adaptation from standing genetic variation is approximately [22]

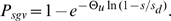
(B2)Under the above scenario, *P_sgv_* is very low even in the case of extremely strong positive selection (*P_sgv_*∼1% for *s*∼100%).

Let us now consider *de novo* resistant mutations that arise after the onset of pesticide application. The average time it takes for an adaptive mutation to emerge and to reach sufficiently high frequency, *x*∼1/(4*N_e_s*), assuring its escape from initial stochastic loss, the so-called establishment time *T_e_*, is on the order of [34], [48]

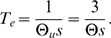
(B3)Once established, the frequency trajectory *x*(*t*) of the adaptive mutation becomes essentially deterministic and can be modelled by [48]

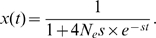
(B4)From establishment it takes on the order of
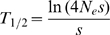
(B5)generations for the mutation to rise to intermediate population frequency (∼50%). The overall expected waiting time *T_w_* for a *de novo* adaptive mutation to reach intermediate frequencies is then
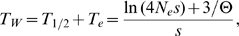
(B6)which is Equation (1) in the main text.

The probability of successful adaptation from *de novo* mutations depends on the expected waiting time for an adaptive mutation to emerge and to reach substantial frequencies in the population. This waiting time is the sum of the expected times to complete two distinct phases: (1) the establishment phase in which an adaptive mutation arises and reaches the frequency at which its escape from stochastic loss is assured and (2) the sweep phase in which the adaptive allele reaches an intermediate population frequency such that it can be readily observed. In Box 1 we show that the overall waiting time can be estimated as
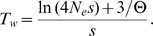
(1)


This equation implies that selection must already be very strong for a single 1-mutation allele to arise and to become prevalent in less than 1500 generations (*s*>20% for Θ = 0.01). Selection coefficients associated with the 2-mutation and 3-mutation alleles need to be even stronger given that they have to outcompete the 1-mutation and 2-mutation alleles respectively.

We have established that under this simple model if Θ is 0.01, the adaptation at *Ace* likely involved very strong positive selection acting on *de novo* mutations. Can we then explain the second empirical observation, namely that the same adaptive mutation by state is observed on several haplotypes that differ in the immediate vicinity of the adaptive site?

We can imagine two scenarios that would generate this observation. In the first, the so called hard sweep scenario, a single adaptive mutation arises in frequency in the population and eventually ends up on different haplotypes due to recombination or mutation events that take place in its vicinity during the sweep. In the other, an example of the so-called soft sweep scenario, several independent adaptive mutations take place on different haplotypes and increase in frequency simultaneously.

Theoretical investigations under simple scenarios by Pennings and Hermisson [20]–[22] showed that such soft sweeps are extremely uncommon if Θ per site is on the order of 0.01 independently of the strength of positive selection. The probability of the hard sweep scenario resulting in the observation of the haplotypic diversity in the vicinity of the adaptive allele is calculated in Box 2. Specifically, we demonstrate that the probability *P_d_* that at least two haplotypes are observed at the end, where the minor haplotype is present in at least a fraction *d* of the population, is approximately
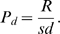
(2)

**Box 2. Probability of distinct haplotypes in a hard sweep.** Consider a single adaptive mutation that reaches establishment frequency in generation *t* = 0. Its subsequent frequency trajectory is *x*_1_(*t*). Mutated or recombined variants of its original haplotype become established in the population at rate
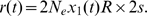
(B7)Here *R* is the rate of either mutation or recombination taking place on the sweeping initial haplotype per individual per generation, and the factor 2*s* is the probability of an adaptive mutation to escape the initial stochastic loss. Note that this is an overestimate of the establishment probability of the second haplotype. If *x*_1_ is substantial in frequency, establishment occurs with a probability that is closer to 2*s*(1−*x*_1_).

What is the probability that a mutated or recombined haplotype also reaches at least frequency *d* in the population? Such a second haplotype has to emerge within a limited number *T_d_* of generations after the first. Otherwise the population will already be dominated by the first haplotype, neutralizing any selective advantage of the second.

Let us assume that the second haplotype becomes established at time *T_d_*. We denote its frequency trajectory by *x*_2_(*t*). The crucial observation allowing us to calculate *T_d_* is that the ratio *x*_1_(*t*)/*x*_2_(*t*) remains constant for all *t*≥*T_d_*, as both haplotypes have the same fitness. In particular, because we require that the second haplotype is eventually present in a fraction *d* of the population, we have
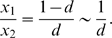
(B8)The latter approximation applies for small *d*≪1. At *t* = *T_d_*, the trajectory *x*_1_(*t*) can still be modelled by Equation (B4); no interference between different adaptive haplotypes has occurred until then because the second allele has been extremely rare or absent for *t*<*T_d_*. Recalling that the second haplotype becomes established when it reaches a frequency *x*_2_∼1/(4*N_e_s*), we thus have:
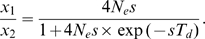
(B9)Solving Equations (B8) and (B9) for *T_d_* and assuming *N_e_sd*≫1 yields
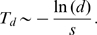
(B10)The condition *N_e_sd*≫1 is justified when positive selection is strong and *d* is large enough that there is a chance of sampling the second allele.

Mutations establishing after *T_d_* can only reach a population frequency smaller than *d*. The probability of observing two different haplotypes with the minor haplotype being present in at least a small fraction *d* of the population can therefore be estimated from the probability that a new variant of the initial adaptive haplotype emerges within the first *T_d_* generations. We obtain
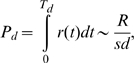
(13)where we again assumed *N_e_sd*≫1. Note that *P_d_* does not depend on Θ.

Here *R* is the total rate of mutation or recombination in the locus per individual per generation and *s* is the strength of positive selection. For our locus of length ∼1500 bp we have *R*∼6*10^−6^ when assuming a recent estimate for the single-site mutation rate in *D.melanogaster* of *m*∼2.5*10^−9^ [23] and a measured recombination rate of *ρ*∼0.15 cM/Mbp [24]. The probability to observe different haplotypes is therefore still very small (*P_d_*<1%) even for a low population frequency of *d* = 2% and assuming *s* to be 5%. Note that this calculation is very conservative given that in our data multiple haplotypes are present at much higher frequency than 2%, multiple haplotypes vary at sites extremely close to the adaptive allele (within 38 bp), and positive selection was likely much stronger.

In conclusion, under this simple scenario, our empirical observations at *Ace* are unexpected if Θ is indeed on the order of 0.01. Specifically, considering how strong selection must be, we should not be seeing more than one distinct haplotype containing the same adaptive mutation.

Note that if Θ were much higher, for example on the order of one or larger, then all of our observations are expected. Soft sweeps would be commonplace because many more mutations enter the population in every generation and can increase in frequency simultaneously thereby generating multiple haplotypes containing the same adaptive mutations [22], as observed in the data. The establishment time would become smaller making it easier to observe complex, 3-mutation alleles at *Ace* in less than 1500 generations. However, selection would still need to be strong because the time it takes for an adaptive allele to reach intermediate frequencies is only weakly (logarithmically) dependent on the effective population size and inversely proportional to the selection coefficient.

### Patterns of evolution at *Ace* require large values of Θ: numerical investigations for a large range of evolutionary scenarios

We have shown above that under very simple population scenarios the pattern of adaptive evolution at *Ace* requires large values of Θ. However, it is unclear whether such large values of Θ are required under more complex and realistic scenarios. Variation in strength of selection, recombination rate, and population structure might affect the probability of evolving complex 3-mutation alleles from the simpler 1- or 2-mutation alleles [25] and the probability of observing multiple haplotypes containing the same adaptive mutations.

To investigate quantitatively the potential impact of such effects we conducted extensive simulations of adaptation at *Ace* under a large number of selective (*s* = 2.5% to 500%) and demographic scenarios (1 to 100 subpopulations, migration rates *M* = 0.01 to 10 individuals per generation between any two subpopulations), and with varying recombination rate (*ρ* = 0 to 10 cM/Mbp) (Table S3).

**Figure 3:**
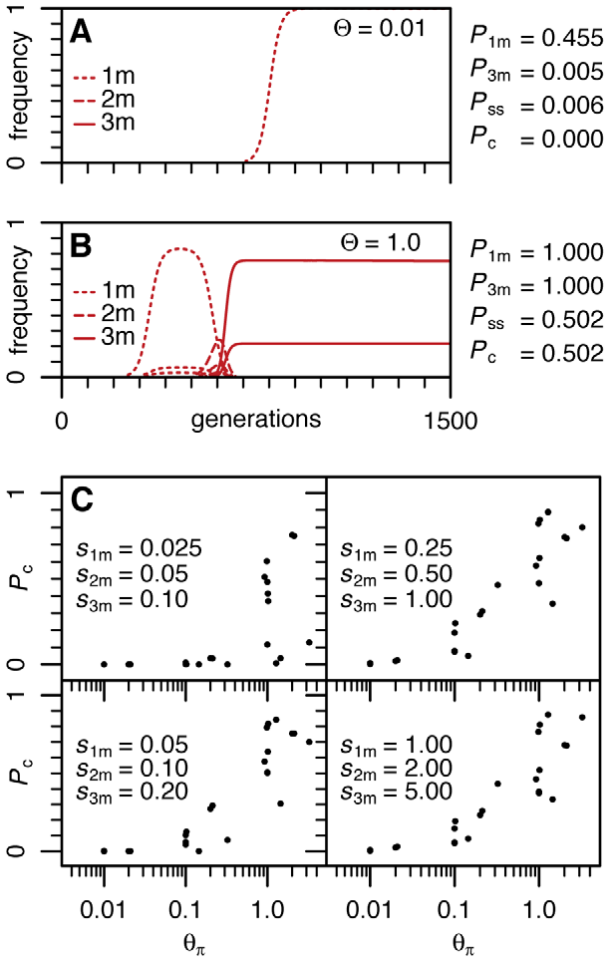
Population dynamics of resistance adaptation for different Θ regimes. (A) Frequency trajectories of resistant haplotypes from a typical simulation of a single population with Θ = 0.01 during the first 1500 generations after pesticides are applied. The selection scenario is *s*_1m_ = 0.05, *s*_2m_ = 0.1, *s*_3m_ = 0.2. Trajectories are shown for all resistant haplotypes that reached a population frequency above 2%. On the right, summary statistics for *P*_1m_, *P*_3m_, *P*_ss_, and *P*_c_ estimated from 10^5^ runs are shown. (B) Frequency trajectories of a typical simulation run and summary statistics for a single population with Θ = 1. This simulation shows a soft sweep for the 3-mutation allele (two different haplotypes at high frequencies; their frequencies together add to 100%). Also, note that 2-mutation alleles do not rise to high frequencies before being taken over by the fitter 3-mutation alleles. (C) *P*_c_ for a variety of different selection, recombination, and population substructure scenarios as specified in Table S3. Probabilities *P*_c_ of each scenario are plotted against the average heterozygosity Θ_π_ of the entire population estimated from coalescent simulations.

In Figure 3A and 3B we show the frequency trajectories of adaptive haplotypes for two representative simulation runs in a simple single population scenario together with summary statistics across a large number of runs for the two key Θ regimes (Θ = 0.01 and Θ = 1). We use four statistics: *P*_1m_ and *P*_3m_ are the probabilities that a single adaptive mutation (1m) allele or the 3-mutation allele (3m) were ever present in at least 10% of the population during the simulation; *P*_ss_ is the probability that a single adaptive mutation is present on distinct haplotypes in a sample of reasonable size (the observation that we will call the soft sweep signature from now on); and *P*_c_ is the combined probability of observing both the complex 3-mutation allele and a single-mutation soft sweep signature during the same simulation. Figure 3A and 3B show results consistent with our analytical considerations. When Θ∼0.01 and selection is of moderate strength, neither the evolution of complex 3-mutation alleles nor soft sweeps signatures are likely. Only when Θ approaches one do both observations become commonplace.

Figure 3C shows the summary of the results for the more complex scenarios (complete results are shown in Table S3). In these more complex scenarios we assessed Θ by using coalescent simulations to estimate the average heterozygosity per site (Θ*_π_*) at neutral sites and by summing Θ across all subpopulations (Θ*_Σ_*) [26]. Our simulations confirm that only when both Θ*_π_* and Θ*_Σ_* become on the order of one or larger is it likely to observe fast evolution of complex 3-mutation alleles and at the same time soft sweep signatures. Strong selection does indeed improve the probability of seeing complex adaptive alleles but also, as expected, does not generate signatures of soft sweeps when Θ is small.

Interestingly our simulations show that if Θ∼1, then most of the observed signatures of soft sweeps are generated by multiple *de novo* mutations and are not due to the recombination of the same adaptive mutation onto different haplotypes. This is because signatures of soft sweeps are still commonly observed in simulations even when the recombination level is set at zero. It is also consistent with analytical considerations under simple scenarios (Text S1).

## Discussion

Our data and analysis strongly suggest that the patterns of adaptation observed at *Ace* in the last 1000–1500 generations are highly unlikely in a population in which Θ per site is on the order of 0.01 as it is commonly assumed. Instead, it appears that Θ per site must have been at least 0.1 and more likely on the order of one or larger. It is possible to elevate Θ by increasing the mutation rate or by increasing the effective population size. We assessed whether *Ace* had an unusually high mutation rate by estimating divergence of *Ace* in *D. melanogaster* from its *D. simulans* ortholog at synonymous sites. We found the divergence to be 7.9%, which is similar to the genome average of ∼10% [27], [28]. In addition, Θ*_π_* per site estimated from polymorphisms at synonymous sites in sensitive alleles is 0.008, which is also consistent with the genome average [18]. Thus we conclude that the effective population size in *D. melanogaster* over the past 1000–1500 generations is likely to be very large (*N_e_*≥10^8^).

Such a large value of *N_e_* might appear puzzling given that levels of standing neutral polymorphism suggest that *N_e_* is much smaller [18], [19]. To resolve this discrepancy it is necessary to take a closer look at the concept of an effective population size. Effective population size is commonly defined by the inverse magnitude of the frequency-fluctuations of a neutral allele in two consecutive generations [1]. Over a number of generations, effective population size is the harmonic mean of the effective population sizes over individual generations and thus is dominated by the smallest values of *N_e_*. (Equivalently, frequency fluctuations over many generations are dominated by the largest fluctuations over single generations). Estimates of the effective population size using frequent neutral polymorphisms reflect *N_e_* harmonically averaged over long periods of time and are therefore very sensitive to any periods of low population size even far back into the past [29].

**Figure 4:**
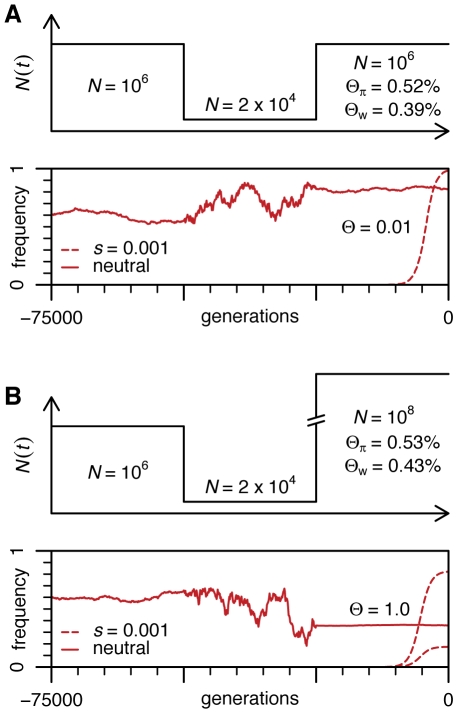
Population dynamics of neutral and adaptive alleles in a population with a bottleneck. (A) The population history similar to that inferred by Thornton and Andolfatto for *D. melanogaster* [18]. Values of Θ_π_ and Watterson's Θ_w_ were obtained from coalescent simulations with 100 sampled genomes. (B) Same scenario as (A) except for the current population size is changed to 10^8^.

In sharp contrast, adaptation at *Ace* occurred within less than 1500 generations. The *N_e_* relevant to adaptation at *Ace* is the harmonic mean of *N_e_* values over the past 1500 generations or even fewer. Unlike *N_e_* measured from ancient standing variation, it is not reduced by the bottlenecks and nearby selective sweeps that occurred more than 1500 generations ago. Consider a simple bottleneck scenario outlined in Figure 4 that is similar to the out-of-Africa scenario of Thornton and Andolfatto [18]. It is apparent that even if the current *N_e_* is 100-fold larger than commonly assumed, population behaviour of a frequent neutral allele does not change substantially and the estimates of Θ from standing variation are not altered. To give another example, if *D. melanogaster* populations were to spend 90% of their time with *N_e_* of 10^10^ and 10% at *N_e_* of 10^5^ with the shifts occurring about every 1000 generations, the harmonic mean *N_e_* derived from common neutral polymorphisms would be ∼10^6^ and yet the adaptive process would take place primarily in populations of 10^10^ with Θ>1 per site. In this case, strong adaptation in *Drosophila* would not be limited by mutation most of the time.

The short-term *N_e_* is bounded by the census population size (*N*) and thus if *N* is much smaller than the reciprocal of the mutation rate per site we can be certain that adaptation would be mutation-limited. In many species *N* can be much larger than the reciprocal of mutation rate and thus in these species it is possible that adaptation is not limited by mutation at single sites. However, it is *N_e_* measured over time scales relevant for adaptation and not *N* that needs to be assessed to answer this question. Even short-term *N_e_* might be much smaller than *N* if populations crash regularly on very fast temporal scales (such as those induced by winters in temperate climates) or if the numbers of successfully reproducing adults in each generation is sharply limited by extrinsic factors, for example by available substrates for laying eggs. Thus the studies of strong adaptation, such as the one presented here, are essential to determining whether adaptation in general is mutation-limited in a species.

It is reasonable that *Drosophila* and many other organisms undergo recurrent boom-bust cycles thereby reducing the long-term *N_e_* strongly but allowing adaptation during the boom years to occur in populations of large short-term *N_e_*. In addition, *Drosophila* appears to undergo pervasive adaptation [30], [31] with most common neutral polymorphisms estimated to have been affected by several selective sweeps in their genomic vicinity [28]. Such pervasive adaptation generates dynamics similar to recurrent bottlenecks and will also reduce the long-term *N_e_* values even if the short-term *N_e_* might be consistently large. This situation is similar to that found in HIV, where the effective population size estimated from observed diversity underestimates the census size by many orders of magnitude and is likely to underestimate the short-term *N_e_* relevant for adaptation as well [32].

The possibility that adaptation at single sites in *D. melanogaster* is not limited by mutation has profound implications. The distinction between standing variation and *de novo* mutations at single sites is blurred since virtually all single-site mutations then exist in the *D. melanogaster* population at any given time. Strong adaptation should be much more rapid and generally result in soft sweeps. Complex adaptations that require multiple changes can be generated without fixation of interim states and with an enhanced chance of crossing fitness valleys [33]. This raises the question of whether the widespread use of the weak mutation, strong selection (“WMSS”) model for the study of adaptation should be broadened to include cases of strong mutation [34], [35].

The number of sweeps (hard or soft) might also in general be lower than the number of adaptive substitutions if complex adaptations requiring multiple substitutions are common. Indeed, in our simulations of evolution at *Ace* in the strong mutation regime (Θ per site on the order of 1), the complex 3-mutation alleles generally evolve without fixation of intermediate 1- and 2-mutation alleles (Figure 3). The number of adaptive substitutions estimated using McDonald-Kreitman approaches should then be larger than the number of independent adaptive fixations and the prediction of the number of selective sweeps derived from the number of adaptive substitutions should be upwardly biased [36].

Note that all of these expectations hold especially well for strong selection because it operates over shorter time scales and is therefore less sensitive to recurrent but infrequent bottlenecks [37] and neighbouring selective sweeps.

Most of the current statistical approaches for the study of adaptation rely on the expected signatures of hard sweeps [30]. Such methods should regularly miss or misidentify strong adaptation if it in fact commonly involves soft sweeps as in the case of *Ace* [20]. For example, if one searches exclusively for hard sweeps, then complete soft sweeps might appear as ongoing hard sweeps and the polymorphisms associated with the most frequent haplotype would appear as the likeliest candidates for the adaptive mutation whereas the true adaptive mutation would be fixed in the population. Methods exist that have high power to detect soft sweeps [20], but they are used less often because soft sweeps have been considered unlikely *a priori*. However, a number of cases of adaptation in *Drosophila* and mosquitoes show clear signatures of soft sweeps [38]–[40]. Soft sweeps might also be common in humans, with the soft sweep associated with lactase persistence providing the strongest signature of adaptation in humans [41], [42]. Our results suggest that the possibility of pervasive soft sweeps needs to be taken seriously.

Recurrent boom-bust cycles are a general feature in population dynamics of most studied organisms. Adaptation and recurrent selective sweeps reducing the long-term but not the short-term *N_e_* might also be common. It follows then that short-term and long-term *N_e_* values are likely to be different as a rule. The shortest term *N_e_* is only bounded by the census population size, which is often very large and can easily be in the billions, particularly for insects or marine organisms. It is thus possible that strong adaptation at single sites may not be limited by mutation in many eukaryotes, similar to the situation found in bacteria and viruses [32].

## Materials and Methods

### *Ace* locus genotyping

We sequenced 1450 bp encompassing exons 2 through 4 of *Ace*. Resistant mutations I161V and G265A lie in the 3rd exon while F330Y and G368A lie in the 4th exon (Figure S1). Initially we sequenced this locus in 68 strains from 20 populations chosen to represent the *Ace* locus in a variety of geographical locations. The list of the populations and the number of lines investigated are given in Table S1 and Table S2. For some of the strains that appeared heterozygous after sequencing of the PCR product, the DNA was first amplified using a proofreading DNA polymerase (Platinum Pfx; INVITROGEN) and cloned using Zero Blunt TOPO PCR cloning kit (INVITROGEN) before sequencing. Note that not all heterozygous strains were cloned, only those that contained a resistant mutation and the AF strains. The primers used for PCR amplification of the *Ace* locus were:

Ace1F: gctggttagtttgccgtaat
Ace1R: ccatgatatccgcattgtaga
Ace2F: aatccgcagaacacgaccaac
Ace2R: cgtgagcgggattggtct
Ace3F: gccttaacgcgtcactcac
Ace3R: aagcttggcaaacaacattgg

PCR products were then sequenced. Of the 68 sequenced strains, 26/68 (∼40%) have a single or multiple resistant mutations. Mutations at I161V, G265A and F330Y were identified in isolation and in combination in multiple populations, while G368A was never observed. We then used PASA [43] to identify strains that contained one or more of the three observed mutations and sequenced the identified strains. The primers used for PASA were:

161-F: ccggatcggccaccctggaca
161-R: agtcgttgatcagcgccttgc
265-F: gcgcggaatgatgcagtcggg
265-R: atcaatggtgggcgccgagg
330-F: gaagaggcgcccggcaatgtg
330-R: atggtgggcgccgagggata

The 161 primer pair amplifies more effectively in the presence of the mutation I161V. The 265 primer pair is specific to G265A and the 330 primer pair is specific to G330Y. The annealing temperatures required for allele specific priming used for 161, 265 and 330 were 61.5°C, 59.5°C and 60.6°C respectively. As positive and negative controls we performed PASA on strains in which the resistant sites had been previously characterized. We sequenced 37 strains from 8 populations that had amplified with one or more of the allele-specific primers. 31/37 (84%) of these strains contained resistant mutations. The incorrect classification of the 6 strains is likely due to the addition of excess template to these PCR reactions resulting in non-specific priming. In total, we sequenced the *Ace* locus in 105 strains from 27 populations from five different continents (Table S1). Twelve of these strains were excluded from the analysis due to poor sequence quality.

### Construction of haplotype network

The most parsimonious haplotype network was constructed using TCS 1.21 [44]. All resistant alleles, except those for which we had poor sequence data, and all sensitive alleles observed more than once were used for the construction of the network. All AF strains and M strains were also included in the network to provide information on ancestral and modern variation respectively at the *Ace* locus.

### Estimation of Θ*_π_* and divergence

Measures of Θ*_π_* and divergence with *Drosophila simulans* at the *Ace* locus were obtained using DnaSP [45]. All sensitive strains analyzed in this study were used for the estimation.

### Forward simulations of *Ace* adaptation

Our simulation models the population frequency dynamics of haplotypes at the 1.5 kb-long sequenced *Ace* locus and incorporates mutation, recombination, selection, and population substructure.

Haplotypes are classified by their particular adaptive allele configuration at the three adaptive sites. We describe this configuration in terms of a vector *a*_1_ *a*_2_ *a*_3_, indicating whether at site *i* the resistance-conferring mutation is present (*a_i_* = 1) or not (*a_i_* = 0). A configuration 101, for example, specifies resistant mutations at sites one and three, but no resistant mutation at site two.

We use an infinite alleles model for new haplotypes, *i.e.* every mutation or recombination event at the locus is assumed to give rise to a new haplotype, which can be distinguished from all other haplotypes in the population. This is implemented in our simulations by assigning a unique ID to every new haplotype. The specific nucleotide sequence of the new haplotype is not relevant for our purposes; only changes in the adaptive-allele configuration are modelled explicitly. We also do not distinguish different sensitive haplotypes as we focus on the population dynamics of adaptive haplotypes. These simplifications substantially increase the performance of our simulations, allowing us to investigate scenarios with population sizes up to 10^9^ in reasonable run-time.

Mutations at adaptive sites and recombination events where the recombination breakpoint lies between two adaptive sites can generate new haplotypes with different adaptive-allele configuration (Table S4). Note that at each site only one specific nucleotide is the resistant allele and thus only one out of three mutations of a sensitive allele will give rise to it.

The evolution of haplotype frequencies is simulated in terms of a Wright-Fisher model with directional selection, *i.e.* we assume panmictic subpopulations of constant size and non-overlapping generations [46]. Every haplotype *h* has a specific selection coefficient *s*(*h*). The mean fitness of a subpopulation at time *t* is 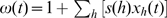, where *x_h_*(*t*) is the frequency of haplotype *h* in the subpopulation at time *t*. Haplotype frequencies in generation *t*+1 are obtained by sampling from a multinomial distribution *B*(2*N*,{*p_h_*}) with selection-adjusted probabilities 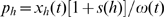.

We group resistant haplotypes into three classes according to the number of resistance-conferring mutations they bear: 1m haplotypes have one resistant allele (100,010,001), 2m haplotypes have two (011,101,110), and 3m haplotypes have all three resistant alleles (111). For simplicity, we assume that all haplotypes in the same class have equal selection coefficients *s*_1m_, *s*_2m_, and *s*_3m_, respectively. Prior to pesticide application all resistant haplotypes are modelled to be deleterious with selection coefficient −*s*_1m_.

The key simulation parameters are the selection scenario defined by the selection coefficients *s*_1m_, *s*_2m_, and *s*_3m_, the recombination rate *ρ*, the number *n* of subpopulations, the migration rate *M* between subpopulations, and the value of Θ within subpopulations. We use a constant mutation rate of *μ* = 2.5 * 10^−9^ per site per generation [23]. Different Θ-values thus correspond to different subpopulation sizes. In particular, Θ = 0.01 corresponds to *N* = 10^6^, and Θ = 1.0 corresponds to *N* = 10^8^. We estimated a recombination rate of *ρ* = 0.15 cM/Mbp for our locus [24], but investigate also other recombination rates in our simulations.

Simulation runs start with one single sensitive haplotype present in all subpopulations at 100% frequency. Before pesticide application commences, mutation-selection equilibrium of resistant haplotypes is established within a burn-in period of 1000 generations. This fully suffices to establish equilibrium due to the strong purifying selection against all resistant haplotypes prior to pesticide application (Box 1). We also verified that longer burn-in times do not change our results. After the burn-in period, pesticide application starts by switching to the corresponding selection scheme. The simulation is then followed for another 1500 generations representing approximately 50 years of pesticide usage. During every generation individual subpopulations evolve according to the following steps:

1. A random number of mutation events is drawn from a Poisson distribution with mean *μ* * 1.5 kb * 2*N*. For each mutation a random haplotype is drawn from the subpopulation and mutated at a randomly chosen position.
2. A random number of recombination events is drawn from a Poisson distribution with mean *ρ* * 10^−8^ * 1.5 kb * 2*N*. For each recombination event two random haplotypes are drawn from the subpopulation and recombined at a randomly chosen breakpoint.
3. The numbers of migrating individuals to each other subpopulation are drawn from a Poisson distribution with mean *M*. For each migrating individual two random haplotypes are drawn from the source population and added to the destination subpopulation.
4. All haplotype frequencies are evolved one generation according to the above-described binomial sampling procedure.

During a simulation run we analyze whether resistant haplotypes emerged and whether soft sweep signatures among 1m haplotypes were observed. We define 1m resistance by at least one of the three 1m adaptive-allele configurations (001, 010, or 100) ever being present in more than 10% of the population during the run. Accordingly, 3m resistance is defined by the complex 3-mutation allele (111) ever present in at least 10% of the population. A soft sweep signature (ss) is ascertained if at any time during the run two independently drawn alleles have greater than 10% probability to bear the same 1m configuration on different haplotypes. The statistics *P*_1m_, *P*_3m_, and *P*_ss_ are the respective probabilities averaged over many runs. *P*_c_ denotes the combined probability that 3m resistance emerged and a soft sweep signature was observed during the same run.

A crucial assumption of our simulation is the applicability of an infinite alleles model, *i.e.* all mutation or recombination events are assumed to be detectable. This can lead to an overestimation of the probabilities to observe soft sweep signatures in our simulations if independent mutation events frequently occur on the same haplotype, or if newly recombined haplotypes often resemble haplotypes already present in the population. We can estimate the resulting error from the probability that an individual is homozygous for the 1.5 kb-long locus. From coalescent simulations using *ms* [26] we infer it to be on the order of ∼10% when assuming a per site heterozygosity of out-of-Africa *D. melanogaster* subpopulations of Θ*_π_*∼0.5% [18], [19] and the above specified recombination and mutation rates for our locus. Note, however, that in any case the infinite alleles model can only lead to an overestimation of the probability to observe soft sweep signatures. It is therefore always conservative in terms of our analysis. The probabilities *P*_1m_ and *P*_3m_ are not affected by the choice of an infinite alleles model.

The simulation was implemented in C++. Runs were performed on the Bio-X2 cluster at Stanford University. All source code is available from the authors upon request.

## Supporting Information

Figure S1 Structure of the *Ace* gene.
(0.04 MB PDF)

Table S1 Description of *D. melanogaster* strains.
(0.05 MB PDF)

Table S2 Segregating sites at the *Ace* locus in all strains sequenced.
(0.07 MB PDF)

Table S3 Dynamics of resistance adaptation for different population parameters.
(0.06 MB PDF)

Table S4 Change of mutation configuration due to mutation or recombination.
(0.04 MB PDF)

Text S1 Origin of soft sweep signatures.
(0.11 MB PDF)
