# Supplementary figures and images for: Evidence that Adaptation in Drosophila Is Not Limited by Mutation at Single Sites
Source: PLoS Genet. 2010 Jun 17;6(6):e1000924. doi: 10.1371/journal.pgen.1000924 (PMC2887467; doi:10.1371/journal.pgen.1000924)

**Figure S1. Structure of the *Ace* gene.**

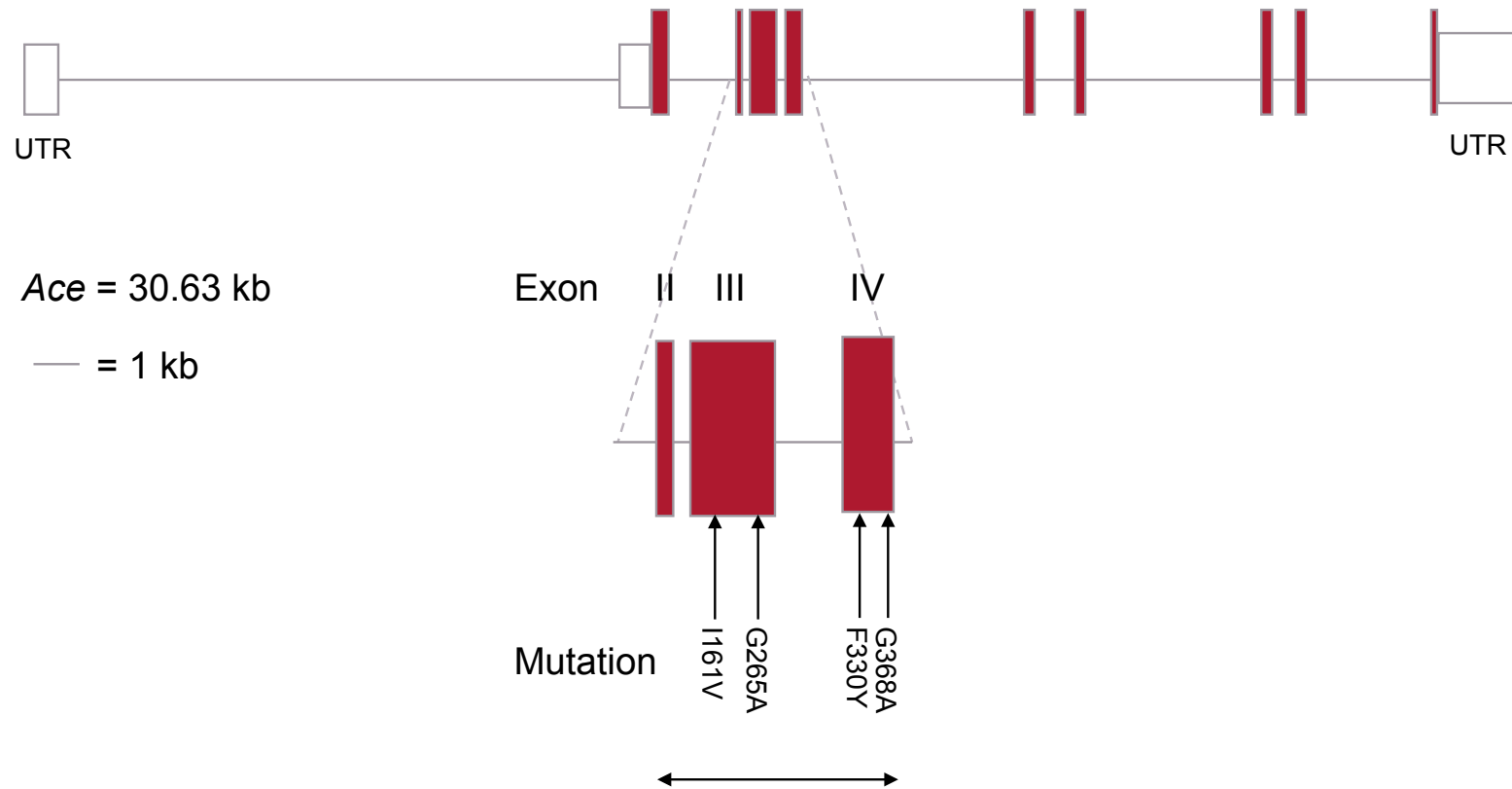

Sequenced 1.5 kb of *Ace* encompassing exons II-IV

Supplement: Figure S1 — Structure of the Ace gene. (0.04 MB PDF) [file pgen.1000924.s001.pdf]
