# Supplementary material for: Evidence that Adaptation in Drosophila Is Not Limited by Mutation at Single Sites
Source: PLoS Genet. 2010 Jun 17;6(6):e1000924. doi: 10.1371/journal.pgen.1000924 (PMC2887467; doi:10.1371/journal.pgen.1000924)
Supplement: Table S1 — Description of D. melanogaster strains. (0.05 MB PDF) [file pgen.1000924.s002.pdf]

**Table S1. Description of *D. melanogaster* strains.**		
Name of population	Geographical origin	Reference
Wi	Wolfskill Orchard, Davis, CA, USA	Sergey Nuzhdin
We1	Raleigh, NC, USA	Greg Gibson
We2	Raleigh, NC, USA	Greg Gibson
Bak	Bakersfield, CA, USA	Dmitri Petrov
Med	Medford, OR, USA	Dmitri Petrov
SD	San Diego, CA, USA	Peter Andolfatto
WMG	Fort Pierce, FL, USA	Paul Schmidt
RR06	Bowdoinham, ME, USA	Paul Schmidt
Por	Portland, OR, USA	Dmitri Petrov
ZH	Zimbabwe	Peter Andolfatto
ZS	Zimbabwe	Peter Andolfatto
ZW	Zimbabwe	Peter Andolfatto
Ky	Kenya	Peter Andolfatto
N	Queensland, Australia	Ary Hoffmann
S	Victoria, Australia	Ary Hoffmann
MAE	Redland Bay, Queensland, AUS	Ary Hoffmann
M	Melbourne, Victoria, AUS	Ary Hoffmann
Amherst-3	-	M strain
Berlin-K	-	M strain
Canton-S	-	M strain
Csiso-3	-	M strain
Lausanne-S	-	M strain
Oregon R-C	-	M strain
Oregon-R-S	-	M strain
Oriso-2	-	M strain
Samarkand	-	M strain
Swedish-C	-	M strain
