# Supplementary material for: Evidence that Adaptation in Drosophila Is Not Limited by Mutation at Single Sites
Source: PLoS Genet. 2010 Jun 17;6(6):e1000924. doi: 10.1371/journal.pgen.1000924 (PMC2887467; doi:10.1371/journal.pgen.1000924)
Supplement: Table S2 — Segregating sites at the Ace locus in all strains sequenced. (0.07 MB PDF) [file pgen.1000924.s003.pdf]

Table S2. Segregating sites at the *Ace* locus in all strains sequenced.

AA change																					I161V														G265A																						F330Y						
nt position		14467	14470	14506	14565	14566	14587	14593	14600	14610	14661	14665	14678	14680	14695	14703	14714	14735	14755	14756	14870	14879	14887	14908	14932	14933	14946	14980	14983	14992	15133	15146	15154	15169	15183	15187	15244	15291	15301	15314	15316	15343	15365	15368	15384	15386	15394	15409	15415	15423	15425	15436	15457	15482	15515	15517	15537	15567	15600	15615	15626	15641	15671
y[1];cn[1]bw[1]sp[1]		T	T	C	C	A	G	T	G	T	A	A	T	A	G	A	A	T	G	G	A	G	C	A	G	G	G	A	C	G	C	A	T	A	G	T	G	A	T	A	G	T	T	C	A	A	A	A	A	G	A	A	C	G	T	C	T	C	C	A	C	A	A
Fig.1 label	strain name																																																														
w-1	wi1	.	.	.	.	.	T	.	.	.	.	C	.	.	.	.	G	.	.	.	G	.	.	.	.	.	.	.	.	.	.	.	.	.	C	.	.	.	.	.	.	A	.	.	.	.	.	.	.	.	.	.	.	.	.	.	A	.	.	.	.	.	.
w-2	wi3	.	.	.	.	.	T	.	.	.	.	C	.	.	.	.	G	.	.	.	G	.	.	.	.	.	.	.	.	.	.	.	.	.	C	.	.	.	.	.	.	A	.	.	.	.	.	.	.	.	.	.	.	.	.	.	A	.	.	.	.	.	.
w-3	wi83	.	.	.	.	.	T	.	.	.	.	C	.	.	.	.	G	.	.	.	G	.	.	.	.	.	.	.	.	.	.	.	.	.	C	.	.	.	.	.	.	A	.	.	.	.	.	.	.	.	.	.	.	.	.	.	A	.	.	.	.	.	.
w-4	we11	.	.	.	.	.	T	.	.	.	.	C	.	.	.	.	G	.	.	.	G	.	.	.	.	.	.	.	.	.	.	.	.	.	C	.	.	.	.	.	.	A	.	.	.	.	.	.	.	.	.	.	.	.	.	.	A	.	.	.	.	.	.
w-5	we88	.	.	.	.	.	T	.	.	.	.	C	.	.	.	.	G	.	.	.	G	.	.	.	.	.	.	.	.	.	.	.	.	.	C	.	.	.	.	.	.	A	.	.	.	.	.	.	.	.	.	.	.	.	.	.	A	.	.	.	.	.	.
w-6	Por10	.	.	.	.	.	T	.	.	.	.	C	.	.	.	.	G	.	.	.	G	.	.	.	.	.	.	.	.	.	.	.	.	.	C	.	.	.	.	.	.	A	.	.	.	.	.	.	.	.	.	.	.	.	.	.	A	.	.	.	.	.	.
v-1	wi77	.	.	.	.	.	.	.	.	.	.	.	.	.	.	.	.	.	.	.	G	.	.	.	.	.	.	.	.	.	.	.	.	.	C	.	.	.	.	.	.	A	.	.	.	.	.	.	.	.	.	.	.	.	.	.	A	.	.	.	.	.	.
v-2	we10	.	.	.	.	.	.	.	.	.	.	.	.	.	.	.	.	.	.	.	G	.	.	.	.	.	.	.	.	.	.	.	.	.	C	.	.	.	.	.	.	A	.	.	.	.	.	.	.	.	.	.	.	.	.	.	A	.	.	.	.	.	.
v-3	we44	.	.	.	.	.	.	.	.	.	.	.	.	.	.	.	.	.	.	.	G	.	.	.	.	.	.	.	.	.	.	.	.	.	C	.	.	.	.	.	.	A	.	.	.	.	.	.	.	.	.	.	.	.	.	.	A	.	.	.	.	.	.
v-4	we67	.	.	.	.	.	.	.	.	.	.	.	.	.	.	.	.	.	.	.	G	.	.	.	.	.	.	.	.	.	.	.	.	.	C	.	.	.	.	.	.	A	.	.	.	.	.	.	.	.	.	.	.	.	.	.	A	.	.	.	.	.	.
v-5	we91	.	.	.	.	.	.	.	.	.	.	.	.	.	.	.	.	.	.	.	G	.	.	.	.	.	.	.	.	.	.	.	.	.	C	.	.	.	.	.	.	A	.	.	.	.	.	.	.	.	.	.	.	.	.	.	A	.	.	.	.	.	.
v-6	Por2	.	.	.	.	.	.	.	.	.	.	.	.	.	.	.	.	.	.	.	G	.	.	.	.	.	.	.	.	.	.	.	.	.	C	.	.	.	.	.	.	A	.	.	.	.	.	.	.	.	.	.	.	.	.	.	A	.	.	.	.	.	.
v-7	Med8	.	.	.	.	.	.	.	.	.	.	.	.	.	.	.	.	.	.	.	G	.	.	.	.	.	.	.	.	.	.	.	.	.	C	.	.	.	.	.	.	A	.	.	.	.	.	.	.	.	.	.	.	.	.	.	A	.	.	.	.	.	.
v-8	Bak3	.	.	.	.	.	.	.	.	.	.	.	.	.	.	.	.	.	.	.	G	.	.	.	.	.	.	.	.	.	.	.	.	.	C	.	.	.	.	.	.	A	.	.	.	.	.	.	.	.	.	.	.	.	.	.	A	.	.	.	.	.	.
v-9	SD3	.	.	.	.	.	.	.	.	.	.	.	.	.	.	.	.	.	.	.	G	.	.	.	.	.	.	.	.	.	.	.	.	.	C	.	.	.	.	.	.	A	.	.	.	.	.	.	.	.	.	.	.	.	.	.	A	.	.	.	.	.	.
v-10	N5	.	.	.	.	.	.	.	.	.	.	.	.	.	.	.	.	.	.	.	G	.	.	.	.	.	.	.	.	.	.	.	.	.	C	.	.	.	.	.	.	A	.	.	.	.	.	.	.	.	.	.	.	.	.	.	A	.	.	.	.	.	.
v-11	N48	.	.	.	.	.	.	.	.	.	.	.	.	.	.	.	.	.	.	.	G	.	.	.	.	.	.	.	.	.	.	.	.	.	C	.	.	.	.	.	.	A	.	.	.	.	.	.	.	.	.	.	.	.	.	.	A	.	.	.	.	.	.
v-12	N63	.	.	.	.	.	.	.	.	.	.	.	.	.	.	.	.	.	.	.	G	.	.	.	.	.	.	.	.	.	.	.	.	.	C	.	.	.	.	.	.	A	.	.	.	.	.	.	.	.	.	.	.	.	.	.	A	.	.	.	.	.	.
v-13	MAE13	.	.	.	.	.	.	.	.	.	.	.	.	.	.	.	.	.	.	.	G	.	.	.	.	.	.	.	.	.	.	.	.	.	C	.	.	.	.	.	.	A	.	.	.	.	.	.	.	.	.	.	.	.	.	.	A	.	.	.	.	.	.
v-14	MAE15	.	.	.	.	.	.	.	.	.	.	.	.	.	.	.	.	.	.	.	G	.	.	.	.	.	.	.	.	.	.	.	.	.	C	.	.	.	.	.	.	A	.	.	.	.	.	.	.	.	.	.	.	.	.	.	A	.	.	.	.	.	.
v-15	MAE22	.	.	.	.	.	.	.	.	.	.	.	.	.	.	.	.	.	.	.	G	.	.	.	.	.	.	.	.	.	.	.	.	.	C	.	.	.	.	.	.	A	.	.	.	.	.	.	.	.	.	.	.	.	.	.	A	.	.	.	.	.	.
p-1	wi18	.	.	.	.	.	.	.	.	.	.	.	.	.	.	.	.	.	.	.	G	.	.	.	.	.	.	.	.	.	.	.	.	.	.	.	.	.	.	.	.	.	.	.	.	.	.	.	.	.	.	.	.	.	.	.	.	.	.	.	.	.	.
p-2	wi45	.	.	.	.	.	.	.	.	.	.	.	.	.	.	.	.	.	.	.	G	.	.	.	.	.	.	.	.	.	.	.	.	.	.	.	.	.	.	.	.	.	.	.	.	.	.	.	.	.	.	.	.	.	.	.	.	.	.	.	.	.	.
p-3	wi98	.	.	.	.	.	.	.	.	.	.	.	.	.	.	.	.	.	.	.	G	.	.	.	.	.	.	.	.	.	.	.	.	.	.	.	.	.	.	.	.	.	.	.	.	.	.	.	.	.	.	.	.	.	.	.	.	.	.	.	.	.	.
p-4	we7	.	.	.	.	.	.	.	.	.	.	.	.	.	.	.	.	.	.	.	G	.	.	.	.	.	.	.	.	.	.	.	.	.	.	.	.	.	.	.	.	.	.	.	.	.	.	.	.	.	.	.	.	.	.	.	.	.	.	.	.	.	.
p-5	we47	.	.	.	.	.	.	.	.	.	.	.	.	.	.	.	.	.	.	.	G	.	.	.	.	.	.	.	.	.	.	.	.	.	.	.	.	.	.	.	.	.	.	.	.	.	.	.	.	.	.	.	.	.	.	.	.	.	.	.	.	.	.
q	Por12	.	.	.	.	.	.	.	.	.	.	.	.	.	.	.	.	.	.	.	G	.	.	.	.	.	.	.	.	.	.	.	.	.	.	.	.	.	.	.	.	.	.	.	.	.	.	.	.	.	.	.	.	.	.	.	.	.	.	.	.	.	G
r	Por21	.	.	.	.	.	.	.	.	.	.	.	.	.	.	.	.	.	.	.	G	.	.	.	.	.	.	.	.	.	.	.	.	.	.	A	.	.	.	.	.	.	.	.	.	.	.	.	.	.	.	.	.	.	.	.	.	.	.	.	.	.	.
s	RRo6-11	.	.	.	.	.	.	.	.	.	.	.	.	.	.	.	.	.	.	.	G	-	.	.	.	.	.	.	T	.	.	.	.	.	.	.	.	.	.	.	.	.	.	.	.	.	.	.	.	.	.	.	.	.	.	.	.	.	.	.	.	.	.
t-1	M5	.	.	G	.	.	.	.	.	.	G	C	.	.	.	.	.	.	.	.	G	.	.	.	.	.	.	.	.	.	.	.	.	.	.	.	.	.	.	.	.	.	.	.	.	.	.	.	.	.	.	.	.	.	.	.	.	.	.	.	.	.	.
t-2	M7	.	.	G	.	.	.	.	.	.	G	C	.	.	.	.	.	.	.	.	G	.	.	.	.	.	.	.	.	.	.	.	.	.	.	.	.	.	.	.	.	.	.	.	.	.	.	.	.	.	.	.	.	.	.	.	.	.	.	.	.	.	.
t-3	M11	.	.	G	.	.	.	.	.	.	G	C	.	.	.	.	.	.	.	.	G	.	.	.	.	.	.	.	.	.	.	.	.	.	.	.	.	.	.	.	.	.	.	.	.	.	.	.	.	.	.	.	.	.	.	.	.	.	.	.	.	.	.
t-4	M17	.	.	G	.	.	.	.	.	.	G	C	.	.	.	.	.	.	.	.	G	.	.	.	.	.	.	.	.	.	.	.	.	.	.	.	.	.	.	.	.	.	.	.	.	.	.	.	.	.	.	.	.	.	.	.	.	.	.	.	.	.	.
-	M133	.	.	G	.	.	.	.	.	.	G	C	.	.	.	.	.	.	.	.	G	-	-	-	-	-	-	-	-	-	-	-	.	.	.	.	.	.	.	.	.	.	.	.	.	.	.	.	.	.	.	.	.	.	.	.	.	.	.	.	.	.	.
t-5	S27	.	.	G	.	.	.	.	.	.	G	C	.	.	.	.	.	.	.	.	G	.	.	.	.	.	.	.	.	.	.	.	.	.	.	.	.	.	.	.	.	.	.	.	.	.	.	.	.	.	.	.	.	.	.	.	.	.	.	.	.	.	.
t-6	S116	.	.	G	.	.	.	.	.	.	G	C	.	.	.	.	.	.	.	.	G	.	.	.	.	.	.	.	.	.	.	.	.	.	.	.	.	.	.	.	.	.	.	.	.	.	.	.	.	.	.	.	.	.	.	.	.	.	.	.	.	.	.
u	ZS15	.	.	.	.	.	.	.	.	.	.	C	A	T	.	.	.	.	A	.	G	.	.	G	A	.	.	G	.	.	.	.	.	.	.	.	.	.	.	.	.	A	.	.	.	.	.	.	.	.	.	.	T	.	.	.	.	.	.	.	.	.	.
p-6	S48	.	.	.	.	.	.	.	.	.	.	.	.	.	.	.	.	.	.	.	G	.	.	.	.	.	.	.	.	.	.	.	.	.	.	.	.	.	.	.	.	.	.	.	.	.	.	.	.	.	.	.	.	.	.	.	.	.	.	.	.	.	.
p-7	S95	.	.	.	.	.	.	.	.	.	.	.	.	.	.	.	.	.	.	.	G	.	.	.	.	.	.	.	.	.	.	.	.	.	.	.	.	.	.	.	.	.	.	.	.	.	.	.	.	.	.	.	.	.	.	.	.	.	.	.	.	.	.
-	N19	.	.	.	.	.	.	.	.	.	.	.	.	.	.	.	.	.	.	.	.	.	.	.	.	.	.	.	.	.	.	.	.	.	C	.	.	.	.	.	.	.	.	.	.	.	.	.	.	.	.	.	.	.	.	.	.	.	.	.	.	.	.
-	MAE12	.	.	.	.	.	.	.	.	.	.	.	.	.	.	.	.	.	.	.	.	-	.	.	.	.	.	.	.	.	.	.	.	.	C	.	.	.	.	.	.	.	.	.	.	.	.	.	.	.	.	.	.	.	.	.	.	.	.	.	.	.	.
-	N41	.	.	.	.	.	B	.	.	.	.	M	.	.	.	.	.	.	.	.	.	.	.	.	.	.	.	R	.	.	.	.	.	.	S	.	.	.	.	.	.	.	.	.	.	.	.	.	.	.	.	.	.	.	.	.	.	.	.	.	.	.	.
-	ZH23	.	.	.	.	.	.	.	.	.	.	.	.	.	.	.	.	.	.	.	.	.	.	.	.	.	.	.	.	.	.	.	.	.	C	.	.	.	.	.	.	.	.	.	.	.	.	.	.	.	.	.	.	.	.	.	.	.	.	.	.	.	.
-	wi41.5	.	.	.	.	.	.	.	.	.	.	.	.	.	.	.	.	.	.	.	G	.	.	.	.	.	.	.	.	.	.	.	.	.	.	.	.	.	.	.	.	.	.	.	.	.	.	.	.	.	.	.	.	.	.	.	A	.	.	.	.	.	.
-	Bak9	.	.	.	.	.	.	.	.	.	.	.	.	.	.	.	.	.	.	.	G	.	.	.	.	.	.	.	.	.	.	.	.	.	C	.	.	.	.	.	.	.	.	.	.	.	.	.	.	.	.	.	.	.	.	.	.	.	.	.	.	.	.
L	wi68	.	.	.	.	.	.	.	.	.	.	.	.	.	.	.	.	.	.	.	.	.	.	.	.	.	.	.	.	.	.	.	.	.	.	.	.	.	.	.	.	.	.	.	.	.	.	.	.	.	.	.	.	.	.	.	.	.	.	.	.	.	.
H	we4	.	.	.	.	.	T	.	.	.	.	C	.	.	A	.	.	.	.	.	.	.	.	.	A	.	.	G	N	.	.	.	.	.	.	.	.	.	.	.	.	.	.	.	.	.	.	.	.	.	.	.	.	.	C	.	.	.	.	.	.	.	.
I	we13	.	.	.	.	.	T	.	.	.	.	C	.	.	A	.	.	.	.	.	.	.	.	.	.	C	.	G	.	.	.	.	.	.	.	.	.	.	.	.	.	.	.	.	.	.	.	.	.	.	.	.	.	.	.	.	.	.	.	.	.	.	.
H	we17	.	.	.	.	.	T	.	.	.	.	C	.	.	A	.	.	.	.	.	.	.	.	.	A	.	.	G	T	.	.	.	.	.	.	.	.	.	.	.	.	.	.	.	.	.	.	.	.	.	.	.	.	.	.	.	.	.	.	.	.	.	.
I	we25	.	.	.	.	.	T	.	.	.	.	C	.	.	A	.	.	.	.	.	.	.	.	.	.	C	.	G	.	.	.	.	.	.	.	.	.	.	.	.	.	.	.	.	.	.	.	.	.	.	.	.	.	.	.	.	.	.	.	.	.	.	.
-	we33	C	C	.	.	.	.	.	.	.	.	C	.	.	.	.	G	.	.	.	.	.	.	G	A	.	.	G	.	.	.	.	.	.	.	.	.	C	.	.	A	.	.	.	.	.	.	.	C	.	.	.	.	.	.	.	.	.	.	.	.	.	.
-	we37	.	.	.	.	.	.	.	.	.	.	C	.	.	.	T	G	.	.	.	.	.	.	.	.	.	.	G	.	.	.	.	.	C	.	.	.	.	.	.	.	A	.	.	.	.	.	.	.	.	.	.	.	.	.	.	.	.	.	.	A	.	.
H	we50	.	.	.	.	.	T	.	.	.	.	C	.	.	A	.	.	.	.	.	.	.	.	.	A	.	.	G	T	.	.	.	.	.	.	.	.	.	.	.	.	.	.	.	.	.	.	.	.	.	.	.	.	.	.	.	.	.	.	.	.	.	.
-	we57	.	.	.	.	.	T	.	.	.	.	C	.	.	A	.	.	.	.	.	.	.	.	.	.	.	.	G	.	.	.	.	.	.	.	.	.	.	.	.	.	.	.	.	.	.	G	.	.	.	.	.	.	.	.	.	.	.	T	.	.	.	.
I	we60	.	.	.	.	.	T	.	.	.	.	C	.	.	A	.	.	.	.	.	.	.	.	.	.	C	.	G	.	.	.	.	.	.	.	.	.	.	.	.	.	.	.	.	.	.	.	.	.	.	.	.	.	.	.	.	.	.	.	.	.	.	.
J	we63	.	.	.	.	.	T	.	.	.	.	C	.	.	A	.	.	.	.	.	.	.	.	.	.	.	.	G	.	.	.	.	.	.	.	.	.	.	.	.	.	.	.	.	.	.	.	.	.	.	.	.	.	.	.	.	.	.	.	.	.	.	.
H	we70	.	.	.	.	.	T	.	.	.	.	C	.	.	A	.	.	.	.	.	.	.	.	.	A	.	.	G	T	.	.	.	.	.	.	.	.	.	.	.	.	.	.	.	.	.	.	.	.	.	.	.	.	.	.	.	.	.	.	.	.	.	.
H	we80	.	.	.	.	.	T	.	.	.	.	C	.	.	A	.	.	.	.	.	.	.	.	.	A	.	.	G	T	.	.	.	.	.	.	.	.	.	.	.	.	.	.	.	.	.	.	.	.	.	.	.	.	.	.	.	.	.	.	.	.	.	.
-	Med20	.	.	.	.	.	T	.	.	.	.	C	.	.	A	.	.	.	.	.	.	-	N	.	A	.	.	G	T	.	.	.	.	.	.	.	.	.	.	.	.	.	.	.	.	.	.	.	.	.	.	.	.	.	.	.	.	.	.	.	.	.	.
-	SD2	.	.	.	.	.	T	.	.	.	.	C	.	.	A	.	.	.	.	.	.	-	N	.	A	.	.	G	T	.	.	.	.	.	.	.	.	.	.	.	.	.	.	.	.	.	.	.	.	.	.	.	.	.	.	.	.	.	.	.	.	.	.
H	SD11	.	.	.	.	.	T	.	.	.	.	C	.	.	A	.	.	.	.	.	.	-	.	.	A	.	.	G	T	.	.	.	.	.	.	.	.	.	.	.	.	.	.	.	.	.	.	.	.	.	.	.	.	.	.	.	.	.	.	.	.	.	.
-	WMG106	.	.	.	.	.	K	.	.	.	G	C	.	.	R	.	.	.	.	.	.	-	S	.	.	.	.	G	.	.	.	.	Y	.	.	.	.	.	.	.	.	N	.	.	.	.	.	.	.	.	.	.	.	.	.	.	.	.	.	.	.	.	-
-	WMG109	.	.	.	.	.	K	.	.	.	G	C	.	.	R	.	.	.	.	.	.	-	.	.	.	A	.	G	T	.	.	.	.	.	.	.	.	.	.	.	.	.	.	A	G	.	.	.	.	T	.	.	.	.	.	.	.	M	.	.	.	.	.
-	WMG115	.	.	.	.	.	T	.	.	.	.	C	.	.	A	.	.	.	.	.	.	-	-	.	A	.	.	G	T	.	.	.	.	.	.	.	.	.	.	.	.	.	.	.	R	.	.	-	-	-	-	-	.	.	.	.	.	.	.	.	.	.	.
-	WMG119	.	.	.	.	.	.	.	.	.	G	C	.	.	.	.	.	.	.	.	.	-	S	.	.	C	.	G	.	.	.	.	.	.	.	.	.	.	.	.	.	.	.	.	.	.	.	.	.	.	.	.	.	.	.	.	.	.	.	.	.	.	.
-	WMG123	.	.	.	.	.	.	.	.	.	.	.	.	.	.	.	.	.	.	.	.	-	.	.	R	.	.	.	.	.	.	.	.	.	.	.	T	.	Y	.	.	.	.	.	.	.	.	.	.	.	.	.	.	.	.	.	.	.	.	.	.	.	.
-	N38	.	.	.	.	.	.	.	.	.	.	.	.	.	.	.	.	.	.	.	.	.	.	.	.	.	.	G	.	.	.	.	.	.	.	.	.	.	.	.	.	.	.	.	.	.	.	.	.	.	.	.	.	.	.	.	.	.	.	.	.	.	.
M / N	N32	.	.	G	.	.	.	.	.	.	G	C	.	.	.	.	.	.	.	.	.	.	.	.	.	.	.	N	.	.	.	.	.	.	.	.	.	.	.	.	.	.	.	.	.	.	.	.	.	.	.	.	.	.	.	.	.	.	.	.	.	.	.
-	N52	.	.	G	.	.	.	.	.	.	G	.	.	.	.	.	.	.	.	.	.	-	.	.	.	.	.	.	.	.	.	.	.	.	.	.	.	.	.	.	.	.	.	.	.	.	.	.	.	.	.	.	.	.	.	.	.	.	.	.	.	.	.
-	MAE15	.	.	G	.	.	.	.	A	.	G	C	.	.	.	.	.	.	.	.	.	.	.	.	.	.	.	G	.	.	.	.	.	.	.	.	.	.	.	.	.	.	.	.	.	.	.	.	.	.	.	.	.	.	.	.	.	.	.	.	.	.	.
M	MAE23	.	.	G	.	.	.	.	.	.	G	C	.	.	.	.	.	.	.	.	.	.	.	.	.	.	.	G	.	.	.	.	.	.	.	.	.	.	.	.	.	.	.	.	.	.	.	.	.	.	.	.	.	.	.	.	.	.	.	.	.	.	.
-	M3	.	.	G	.	.	K	.	.	.	G	C	.	.	.	.	.	.	.	.	.	.	.	.	A	.	A	G	Y	.	.	.	.	.	.	.	.	.	.	.	.	.	.	.	.	.	.	.	.	.	.	.	.	.	.	.	.	.	.	.	.	.	.
-	M5	.	.	M	.	.	.	.	.	.	G	C	.	.	.	.	.	.	.	.	.	.	.	.	.	.	.	.	.	.	.	.	.	.	.	.	.	.	.	.	.	.	.	.	.	.	.	.	.	.	.	.	.	.	.	.	.	.	.	.	.	.	.
N	S41	.	.	G	.	.	.	.	.	.	G	C	.	.	.	.	.	.	.	.	.	.	.	.	.	.	.	.	.	.	.	.	.	.	.	.	.	.	.	.	.	.	.	.	.	.	.	.	.	.	.	.	.	.	.	.	.	.	.	.	.	.	.
-	S44	.	.	.	.	.	T	.	.	.	.	C	.	.	A	.	.	.	.	.	.	.	.	.	N	.	.	N	N	.	.	.	.	.	.	.	.	.	.	.	.	.	.	.	.	.	.	.	.	.	.	.	.	.	.	.	.	.	.	.	.	.	.
M / N	S222	.	.	G	.	.	.	.	.	.	G	C	.	.	.	.	.	.	.	.	.	.	.	.	.	.	.	R	.	.	.	.	.	.	.	.	.	.	.	.	.	.	.	.	.	.	.	.	.	.	.	.	.	.	.	.	.	.	.	.	.	.	.
H	Amherst	.	.	.	.	.	T	.	.	.	.	C	.	.	A	.	.	.	.	.	.	.	.	.	A	.	.	G	T	.	.	.	.	.	.	.	.	.	.	.	.	.	.	.	.	.	.	.	.	.	.	.	.	.	.	.	.	.	.	.	.	.	.
L	Berlin-K	.	.	.	.	.	.	.	.	.	.	.	.	.	.	.	.	.	.	.	.	.	.	.	.	.	.	.	.	.	.	.	.	.	.	.	.	.	.	.	.	.	.	.	.	.	.	.	.	.	.	.	.	.	.	.	.	.	.	.	.	.	.
L	Canton-S	.	.	.	.	.	.	.	.	.	.	.	.	.	.	.	.	.	.	.	.	.	.	.	.	.	.	.	.	.	.	.	.	.	.	.	.	.	.	.	.	.	.	.	.	.	.	.	.	.	.	.	.	.	.	.	.	.	.	.	.	.	.
L	Csiso-3	.	.	.	.	.	.	.	.	.	.	.	.	.	.	.	.	.	.	.	.	.	.	.	.	.	.	.	.	.	.	.	.	.	.	.	.	.	.	.	.	.	.	.	.	.	.	.	.	.	.	.	.	.	.	.	.	.	.	.	.	.	.
J	Lausanne-S	.	.	.	.	.	T	.	.	.	.	C	.	.	A	.	.	.	.	.	.	.	.	.	.	.	.	G	.	.	.	.	.	.	.	.	.	.	.	.	.	.	.	.	.	.	.	.	.	.	.	.	.	.	.	.	.	.	.	.	.	.	.
K	Oriso-2	.	.	.	.	.	T	.	.	.	.	C	.	.	A	.	.	.	.	.	.	.	.	.	.	.	.	.	.	.	.	.	.	.	.	.	.	.	.	.	.	.	.	.	.	.	.	.	.	.	.	.	.	.	.	.	.	.	.	.	.	.	.
H	Oregon-R-C	.	.	.	.	.	T	.	.	.	.	C	.	.	A	.	.	.	.	.	.	.	.	.	A	.	.	G	T	.	.	.	.	.	.	.	.	.	.	.	.	.	.	.	.	.	.	.	.	.	.	.	.	.	.	.	.	.	.	.	.	.	.
L	Oregon-R-S	.	.	.	.	.	.	.	.	.	.	.	.	.	.	.	.	.	.	.	.	.	.	.	.	.	.	.	.	.	.	.	.	.	.	.	.	.	.	.	.	.	.	.	.	.	.	.	.	.	.	.	.	.	.	.	.	.	.	.	.	.	.
L	Samarkand	.	.	.	.	.	.	.	.	.	.	.	.	.	.	.	.	.	.	.	.	.	.	.	.	.	.	.	.	.	.	.	.	.	.	.	.	.	.	.	.	.	.	.	.	.	.	.	.	.	.	.	.	.	.	.	.	.	.	.	.	.	.
L	Swedish-C	.	.	.	.	.	.	.	.	.	.	.	.	.	.	.	.	.	.	.	.	.	.	.	.	.	.	.	.	.	.	.	.	.	.	.	.	.	.	.	.	.	.	.	.	.	.	.	.	.	.	.	.	.	.	.	.	.	.	.	.	.	.
A	Ky16	.	.	.	.	.	.	.	.	.	.	C	.	.	.	.	.	C	.	A	.	.	.	G	A	.	.	G	.	.	.	.	.	C	.	.	.	.	.	.	.	A	.	.	.	.	.	.	.	.	.	.	T	A	.	A	.	.	.	.	.	.	.
B	Ky26	.	.	.	.	.	.	.	.	.	G	C	.	.	.	.	.	.	.	A	.	.	.	G	A	.	.	G	.	.	.	.	.	C	.	.	.	.	.	.	.	A	.	.	.	.	.	.	.	.	.	.	T	.	.	A	.	.	.	.	.	.	.
F	ZH12	.	.	.	.	.	T	.	.	.	.	C	.	.	A	.	.	.	.	.	.	.	.	.	.	.	.	G	.	.	.	.	.	.	.	.	.	.	.	.	.	.	.	.	.	.	.	T	.	.	G	.	.	.	.	.	.	.	.	.	.	G	.
G	ZH26	.	.	.	.	.	T	.	.	.	.	C	.	.	A	.	.	.	.	.	.	.	.	.	.	.	.	G	.	.	.	.	.	.	.	.	.	.	.	.	.	.	.	.	.	G	.	T	.	.	.	G	.	.	.	.	.	.	.	.	.	.	.
D	ZS15	.	.	.	.	.	T	.	.	.	.	C	.	.	A	.	.	.	.	.	.	.	.	.	.	.	.	G	.	.	.	.	.	.	.	.	.	G	.	.	.	.	.	.	.	.	.	T	.	.	.	.	.	.	.	.	.	.	.	.	.	.	.
E	ZS29	.	.	.	.	.	T	.	.	.	.	C	.	.	A	.	.	.	.	.	.	.	.	.	.	.	.	G	.	.	.	.	.	.	.	.	.	.	.	.	.	.	.	.	.	.	.	T	.	.	.	.	.	.	.	.	.	.	.	.	.	.	.
C	ZW149	.	.	.	.	.	.	.	.	.	G	C	.	.	.	.	.	.	.	.	.	.	.	G	A	.	.	G	.	.	G	.	.	C	.	.	.	.	.	.	.	A	.	.	.	.	.	.	.	.	.	.	T	.	.	.	.	.	.	.	.	.	.
