# Supplementary material for: Evidence that Adaptation in Drosophila Is Not Limited by Mutation at Single Sites
Source: PLoS Genet. 2010 Jun 17;6(6):e1000924. doi: 10.1371/journal.pgen.1000924 (PMC2887467; doi:10.1371/journal.pgen.1000924)
Supplement: Table S3 — Dynamics of resistance adaptation for different population parameters. (0.06 MB PDF) [file pgen.1000924.s004.pdf]

Table S3. Dynamics of resistance adaptation for different population parameters

						s_{1m} = .025, s_{2m} = .05, s_{3m} = .1				s_{1m} = .05, s_{2m} = .1, s_{3m} = .2				s_{1m} = .25, s_{2m} = .5, s_{3m} = 1				s_{1m} = 1, s_{2m} = 2, s_{3m} = 5			
n	Θ	M	ρ	Θ_Σ	Θ_π	P_{1m}	P_{3m}	P_{ss}	P_c	P_{1m}	P_{3m}	P_{ss}	P_c	P_{1m}	P_{3m}	P_{ss}	P_c	P_{1m}	P_{3m}	P_{ss}	P_c
1	0.01	-	0.00	0.01	0.010	0.242	0.000	0.002	0.000	0.455	0.003	0.005	0.000	0.932	0.184	0.008	0.002	0.997	0.552	0.006	0.003
1	0.01	-	0.15	0.01	0.010	0.238	0.000	0.004	0.000	0.455	0.005	0.006	0.000	0.932	0.200	0.008	0.002	0.997	0.563	0.006	0.003
1	0.01	-	1.00	0.01	0.010	0.239	0.000	0.008	0.000	0.456	0.006	0.010	0.000	0.932	0.204	0.010	0.002	0.997	0.564	0.006	0.003
1	0.01	-	10.00	0.01	0.010	0.238	0.001	0.206	0.000	0.456	0.008	0.211	0.003	0.931	0.209	0.040	0.008	0.998	0.568	0.014	0.008
2	0.01	0.01	0.15	0.02	0.145	0.411	0.000	0.020	0.000	0.698	0.015	0.054	0.001	0.995	0.517	0.100	0.050	1.000	0.901	0.091	0.081
2	0.01	1	0.15	0.02	0.021	0.413	0.001	0.018	0.000	0.701	0.024	0.032	0.001	0.995	0.567	0.043	0.024	1.000	0.914	0.033	0.030
2	0.01	10	0.15	0.02	0.020	0.412	0.001	0.015	0.000	0.700	0.027	0.027	0.001	0.995	0.572	0.037	0.020	1.000	0.917	0.027	0.024
10	0.01	0.01	0.15	0.10	0.325	0.853	0.001	0.123	0.000	0.994	0.202	0.437	0.072	1.000	1.000	0.465	0.464	1.000	1.000	0.432	0.432
10	0.01	1	0.15	0.10	0.102	0.894	0.007	0.188	0.001	0.996	0.531	0.241	0.125	1.000	1.000	0.241	0.241	1.000	1.000	0.193	0.193
10	0.01	10	0.15	0.10	0.100	0.909	0.021	0.158	0.003	0.996	0.567	0.181	0.104	1.000	1.000	0.185	0.185	1.000	1.000	0.146	0.146
100	0.01	0.01	0.15	1.00	1.281	1.000	0.014	0.817	0.007	1.000	1.000	0.843	0.843	1.000	1.000	0.889	0.889	1.000	1.000	0.875	0.875
100	0.01	1	0.15	1.00	1.014	1.000	0.696	0.631	0.414	1.000	1.000	0.638	0.638	1.000	1.000	0.622	0.622	1.000	1.000	0.520	0.520
100	0.01	10	0.15	1.00	0.924	1.000	0.918	0.565	0.511	1.000	1.000	0.575	0.575	1.000	1.000	0.577	0.577	1.000	1.000	0.462	0.462
1	0.1	-	0.00	0.10	0.100	0.920	0.001	0.080	0.001	0.998	0.462	0.090	0.043	1.000	1.000	0.079	0.079	1.000	1.000	0.054	0.054
1	0.1	-	0.15	0.10	0.100	0.918	0.056	0.085	0.005	0.997	0.628	0.087	0.054	1.000	1.000	0.077	0.077	1.000	1.000	0.052	0.052
1	0.1	-	1.00	0.10	0.100	0.915	0.097	0.105	0.011	0.998	0.666	0.093	0.059	1.000	1.000	0.074	0.074	1.000	1.000	0.057	0.057
2	0.1	0.01	0.15	0.20	1.450	0.992	0.101	0.323	0.036	1.000	0.874	0.349	0.305	1.000	1.000	0.355	0.355	1.000	1.000	0.333	0.333
2	0.1	1	0.15	0.20	0.213	0.993	0.111	0.305	0.035	1.000	0.921	0.319	0.293	1.000	1.000	0.311	0.311	1.000	1.000	0.259	0.259
2	0.1	10	0.15	0.20	0.201	0.992	0.134	0.287	0.036	1.000	0.936	0.291	0.272	1.000	1.000	0.291	0.291	1.000	1.000	0.232	0.232
10	0.1	0.01	0.15	1.00	3.255	1.000	0.220	0.663	0.128	1.000	1.000	0.700	0.700	1.000	1.000	0.801	0.801	1.000	1.000	0.859	0.859
10	0.1	1	0.15	1.00	1.024	1.000	0.497	0.792	0.370	1.000	1.000	0.817	0.817	1.000	1.000	0.845	0.845	1.000	1.000	0.811	0.811
10	0.1	10	0.15	1.00	0.985	1.000	0.787	0.789	0.603	1.000	1.000	0.793	0.793	1.000	1.000	0.823	0.823	1.000	1.000	0.765	0.765
1	1	-	0.00	1.00	1.000	1.000	0.230	0.515	0.116	1.000	1.000	0.506	0.506	1.000	1.000	0.474	0.474	1.000	1.000	0.373	0.373
1	1	-	0.15	1.00	1.000	1.000	0.964	0.504	0.482	1.000	1.000	0.502	0.502	1.000	1.000	0.475	0.475	1.000	1.000	0.382	0.382
2	1	1	0.15	2.00	2.125	1.000	0.999	0.752	0.751	1.000	1.000	0.755	0.755	1.000	1.000	0.737	0.737	1.000	1.000	0.677	0.677
2	1	10	0.15	2.00	2.012	1.000	0.999	0.757	0.756	1.000	1.000	0.755	0.755	1.000	1.000	0.744	0.744	1.000	1.000	0.681	0.681

Population structure is specified by the number n of subpopulations, Θ within subpopulations, the average number M of individuals that migrate between each pair of subpopulations per generation, and the recombination rate ρ in cM/Mbp. Values of the expected average heterozygosity Θ_π for the entire population were obtained from coalescent simulations with *ms*. Summary statistics are averages over at least 100000 runs for each scenario.
