# Supplementary material for: Evidence that Adaptation in Drosophila Is Not Limited by Mutation at Single Sites
Source: PLoS Genet. 2010 Jun 17;6(6):e1000924. doi: 10.1371/journal.pgen.1000924 (PMC2887467; doi:10.1371/journal.pgen.1000924)
Supplement: Table S4 — Change of mutation configuration due to mutation or recombination. (0.04 MB PDF) [file pgen.1000924.s005.pdf]

Table S4. Change of mutation configuration.

A Mutation:

site \ *h*	000	001	010	100	011	101	110	111
1	100	101	110	000	111	001	010	011
2	010	011	000	110	001	111	100	101
3	001	000	011	101	010	100	111	110

B Recombination:

*h*1 \ *h*2	000	001	010	100	011	101	110	111
000		001 / 000<br>001 / 000	010 / 000<br>000 / 010	000 / 100<br>000 / 100	011 / 000<br>001 / 010	001 / 100<br>001 / 100	010 / 100<br>000 / 110	011 / 100<br>001 / 110
001			010 / 001<br>000 / 011	000 / 101<br>000 / 101	011 / 001<br>001 / 011	001 / 101<br>001 / 101	010 / 101<br>000 / 111	011 / 101<br>001 / 111
010				000 / 110<br>010 / 100	011 / 010<br>011 / 010	001 / 110<br>011 / 100	010 / 110<br>010 / 110	011 / 110<br>011 / 110
100					111 / 000<br>101 / 010	101 / 100<br>101 / 100	110 / 100<br>100 / 110	111 / 100<br>101 / 110
011						001 / 111<br>011 / 101	010 / 111<br>010 / 111	011 / 111<br>011 / 111
101							110 / 101<br>100 / 111	111 / 101<br>101 / 111
110								111 / 110<br>111 / 110

(A) Mutation configuration outcomes for all possible initial configurations *h* of the mutating haplotype (columns) if a mutation occurs at one of the three adaptive sites (rows). White cells denote mutation events that decrease resistance. Gray cells are events that potentially increase resistance. (B) Mutation configuration outcomes for all possible combinations of two recombining haplotypes *h*1, *h*2. The upper line of each cell specifies the mutation configurations of the two resulting haplotypes if the recombination breakpoint lies between sites one and two, lower lines specify the outcome for recombination breakpoints between sites two and three. Notice that recombinations between haplotypes with equal mutation configuration, and events where the recombination breakpoint does not lie between sites one and three, cannot give rise to new configurations. Grey-shaded outcomes represent events where a haplotype with higher resistance than each of the two original haplotypes is generated. The matrix is symmetrical.
