# Supplementary material for: Evidence that Adaptation in Drosophila Is Not Limited by Mutation at Single Sites
Source: PLoS Genet. 2010 Jun 17;6(6):e1000924. doi: 10.1371/journal.pgen.1000924 (PMC2887467; doi:10.1371/journal.pgen.1000924)
Supplement: Text S1 — Origin of soft sweep signatures. (0.11 MB PDF) [file pgen.1000924.s006.pdf]

Text S1. Origin of soft sweep signatures.

Soft sweep signatures, *i.e.* the observation of the same adaptive mutation by state being present on several haplotypes that differ in the immediate vicinity of the adaptive site, can result by two scenarios: (i) In the "hard sweep" scenario, a single adaptive mutation arises in frequency in the population and eventually ends up on different haplotypes due to recombination or mutation events that take place in its vicinity during the sweep. (ii) In the "true soft sweep" scenario several *de novo* adaptive mutations take place on different haplotypes and increase in frequency simultaneously.

Conditional on that we observe a soft sweep signature which, the hard sweep scenario or the true soft sweep scenario was the more likely cause? To calculate the relative likelihood of the two scenarios let us consider again the scenario of a single adaptive locus in a panmictic population of constant size N from Box 1. The possibility of successful adaptation from standing genetic variation has already been rendered highly unlikely in our case. We therefore assume that the first adaptive allele to escape initial stochastic loss emerged at a time $t = 0$ when pesticides were already applied. Note that in both scenarios a second adaptive haplotype needs to emerge while the first is still at low frequency to have a realistic chance of also rising to sufficient population frequency (the threshold time has been calculated in Box 2). We model the adaptive allele's population frequency trajectory $x(t)$ conditional on eventual fixation by

$$x(t) = \frac{1}{1 + 4Ns\,e^{-st}}. \tag{1}$$

While the first adaptive allele is still at low frequency, new adaptive mutations that escape initial loss emerge at rate $u = \Theta s/3$ (Boxes 1 and 2). We describe such events by a Poisson process and assume that different mutations always emerge on different haplotypes. The probability density of waiting times T_u for the next *de novo* adaptive mutation in the true soft sweep scenario is then

$$\Pr(T_u = t) = u\,e^{-ut}. \tag{2}$$

Recombination or mutation on the initial adaptive haplotype in the hard-sweep scenario can be modeled analogously. We again assume that every event gives rise to a new haplotype. If $x(t)$ is still small, the rate $r(t)$ at which recombined or mutated variants of the adaptive haplotype arise is

$$r(t) = 2Nx(t)R \times 2s, \tag{3}$$

where R is the rate of either mutation or recombination taking place on the sweeping haplotype per individual per generation. Notice that $r(t)$ describes a non-stationary Poisson process as its rate depends on the frequency trajectory. The probability $\Pr(T_r > t)$ that the waiting time T_r for a recombined or mutated adaptive haplotype is larger than t is then

$$\Pr(T_r > t) = \exp\left[-\int_0^t r(\tau)\,\mathrm{d}\tau\right] = \left(\frac{4Ns+1}{4Ns+e^{st}}\right)^{4NR}. \quad (4)$$

We can now estimate which of the two scenarios is more likely, by calculating the probability that the waiting time T_u is shorter than the waiting time T_r,

$$\begin{aligned} \Pr(T_u < T_r) &= \int_0^\infty \Pr(T_u = t)\Pr(T_r > t)\,\mathrm{d}t \\ &= \frac{F_1^2(4NR, 4NR+\frac{\Theta}{3}, 1+4NR+\frac{\Theta}{3}, -4Ns)}{(1+4Ns)^{-4NR}(12NR+\Theta)}. \end{aligned} \quad (5)$$

Here $F_1^2(\cdot,\cdot,\cdot,\cdot)$ is the hypergeometric function 2F1. Numerical values of Equation (5) for various population parameters are shown in Supplementary Figure 1. A clear crossover is observed, separating two regimes: For low population sizes, soft sweep signatures primarily originate from recombination or mutation events on the initial adaptive haplotype, *i.e.* the hard sweep scenario; in larger populations, true soft sweeps dominate.

A simple heuristic approximation for the location of the crossover can be derived by comparing the average waiting time $\langle T_u \rangle$ for *de novo* mutation with the average waiting time $\langle T_r \rangle$ for mutation or recombination on the initial adaptive haplotype. For *de novo* mutation, this is simply the inverse of the rate u,

$$\langle T_u \rangle = \frac{3}{\theta s}. \quad (6)$$

The waiting time $\langle T_r \rangle$ can be calculated from $1 = \int_0^{\langle T_r \rangle} r(t)\,\mathrm{d}t$, yielding

$$\langle T_r \rangle = \frac{1}{s}\log\left[4Ns\left(e^{\frac{1}{4NR}} - 1\right)\right] \approx \frac{1}{s}\log\left[s/R\right]. \quad (7)$$

The last approximation holds in the limit $4NR \gg 1$. Crossover between the two regimes implies that $\langle T_u \rangle \approx \langle T_r \rangle$. The corresponding threshold population size is therefore given by

$$N^* = \frac{3}{4\mu\log\left[s/R\right]}. \quad (8)$$

As shown in Supplementary Figure 1, the heuristic threshold is in very good agreement with the actual crossover of Equation (5). For our locus of length 1.5 kb and assuming a mutation rate of $\mu = 2.5 \times 10^{-9}$ per bp and generation and a recombination rate of $\rho = 0.15$ cM/Mbp, we have $R = L(\mu + \rho) = 6 \times 10^{-6}$. For $s = 0.1$ this yields $N^* \approx 3 \times 10^7$. We then clearly expect *de novo* mutations, and hence true soft sweeps, to be the dominant cause of soft sweep signatures in the $\theta \geq 1$ regime for our locus. Note that $1/N^*$ is approximately linear in μ whereas it scales logarithmically in s/R. The location of the crossover is thus primarily a function of the *de novo* mutation rate and depends only weekly on recombination rate and strength of selection.

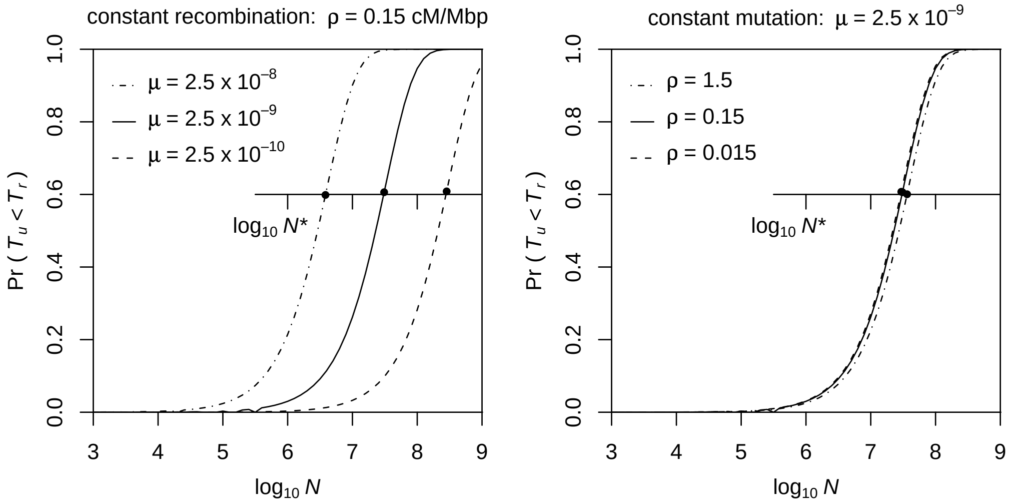

Supplementary Figure 1. Probability that a soft sweep signature originates by a *de novo* adaptive mutation rather than recombination or mutation on the initial adaptive haplotype as a function of population size according to Equation (5) for $s = 0.1$ and $R = 6 \times 10^6$. Dots show the position of the corresponding heuristic cutoff N^* from Equation (8).
